# Dual RNA-Seq Analysis of the Pine-*Fusarium circinatum* Interaction in Resistant (*Pinus tecunumanii*) and Susceptible (*Pinus patula*) Hosts

**DOI:** 10.3390/microorganisms7090315

**Published:** 2019-09-04

**Authors:** Erik A. Visser, Jill L. Wegrzyn, Emma T. Steenkamp, Alexander A. Myburg, Sanushka Naidoo

**Affiliations:** 1 Department of Biochemistry, Genetics and Microbiology, Forestry and Agricultural Biotechnology Institute (FABI), Centre for Bioinformatics and Computational Biology, University of Pretoria, Private Bag X20, Pretoria 0028, South Africa; 2 Department of Ecology and Evolutionary Biology, University of Connecticut, Storrs, CT 06269, USA

**Keywords:** Dual RNA-seq, ergosterol biosynthesis, *Fusarium circinatum*, host-pathogen interaction, *Pinus patula*, *Pinus tecunumanii*

## Abstract

*Fusarium circinatum* poses a serious threat to many pine species in both commercial and natural pine forests. Knowledge regarding the molecular basis of pine-*F. circinatum* host-pathogen interactions could assist efforts to produce more resistant planting stock. This study aimed to identify molecular responses underlying resistance against *F. circinatum*. A dual RNA-seq approach was used to investigate host and pathogen expression in *F. circinatum* challenged *Pinus tecunumanii* (resistant) and *Pinus patula* (susceptible), at three- and seven-days post inoculation. RNA-seq reads were mapped to combined host-pathogen references for both pine species to identify differentially expressed genes (DEGs). *F. circinatum* genes expressed during infection showed decreased ergosterol biosynthesis in *P. tecunumanii* relative to *P. patula*. For *P. tecunumanii*, enriched gene ontologies and DEGs indicated roles for auxin-, ethylene-, jasmonate- and salicylate-mediated phytohormone signalling. Correspondingly, key phytohormone signaling components were down-regulated in *P. patula*. Key *F. circinatum* ergosterol biosynthesis genes were expressed at lower levels during infection of the resistant relative to the susceptible host. This study further suggests that coordination of phytohormone signaling is required for *F. circinatum* resistance in *P. tecunumanii*, while a comparatively delayed response and impaired phytohormone signaling contributes to susceptibility in *P. patula*.

## 1. Introduction

One of the most important pathogens to natural and industrial pine forests is the pitch canker fungus, *Fusarium circinatum* Nirenberg and O’Donnell [1,2,3]. Since the first report of the pathogen, and the disease it causes, in the southeastern US, *F. circinatum* has been identified in more than ten countries world-wide, resulting in significant losses in both nurseries and plantations [2,3,4]. The pathogen was originally identified due to the visible symptom development on pines and classified as a necrotrophic pathogen of pine, capable of infecting douglas fir (*Pseudostuga menziesii*) and up to 60 different species of pine, including economically important species such as *Pinus pinaster*, *Pinus taeda* and *Pinus radiata* [3]. Alarmingly, the last natural stands of *P. radiata* are under threat of extirpation due to pitch canker [5]. Multiple studies, however, have since shown a broader range of potential ecological activities [6]. The pathogen has been shown to endophytically infect pine [7], as well as certain grass species [8], maize [9] and some dicots [10]. Thus, there is a high likelihood for *F. circinatum* to spread into unaffected regions through naturally occurring inoculum reservoirs on unmonitored species [2,7,8,9].

Sterols are important lipids in eukaryotic cellular membranes with vital roles in regulating membrane fluidity and permeability. Ergosterol is present in the membranes of many fungi and is required for fungal growth [11,12]. This sterol has also been shown to play a crucial role in vegetative differentiation and virulence in *Fusarium graminearum* [13]. Plant PR-1 proteins have been shown to affect pathogen growth through binding and sequestration of sterols. While effective against sterol-auxotrophic pathogens such as oomycetes, sterol-prototrophic pathogens such as fungi only become sensitive to PR-1 when their sterol biosynthesis is compromised [14].

The most extensively planted softwood in South African forestry is *Pinus patula*, which is highly susceptible to *F. circinatum* [15]. As a result, cultivation of this species declined by ca. 14% between 2002 and 2016 [16,17] due to high post-planting mortality rates [18,19]. Current long-term control strategies under investigation include the usage of alternative species, breeding and selection programs to produce more resistant families and hybridisation between susceptible and resistant species [15,19]. Commercial deployment of hybrids between *P. patula* and *Pinus tecunumanii* from low elevation provenances, which are resistant to *F. circinatum*, has already started [15,20,21]. Knowledge of the molecular mechanisms underlying host resistance and susceptibility, as well as pathogen virulence, could expedite development of resistant genotypes and improve the effectiveness of genetic resistance.

Ongoing advancements in high-throughput sequencing (HTS) technology and bioinformatics has allowed a more in-depth investigation of transcriptomic responses in non-model plants such as pine. A transcriptome wide analysis in *Pinus monticola* implicated calcium and abscisic acid signaling, as well as down-regulation of photosystems and carbon fixation in resistance to the biotrophic rust fungus *Cronartium ribicola* [22]. A recent transcriptome wide study on *F. circinatum*-challenged *P. radiata* identified increased expression of genes associated with abscisic acid, salicylic acid and ethylene response pathways, in resistant relative to susceptible seedlings [23]. The increasing sensitivity of HTS technologies has also made it feasible to simultaneously sequence expressed genes from both the plant and pathogen in a single sample, an approach referred to as dual RNA-seq, thus allowing parallel investigation of host and pathogen responses during an interaction [24,25,26,27].

In a previous study, transcriptomes were assembled for *P. tecunumanii* and *P. patula* during *F. circinatum* challenge, however, host and pathogen gene expression was not investigated [28]. This study aimed to elucidate molecular mechanisms underlying host resistance by examining pathogen and host responses during *F. circinatum* challenges in pine.

## 2. Results

### 2.1. Annotation

Mercator annotation assigned MapMan functional categories to 34,502 of 52,735 (65%) *P. patula* transcripts and 18,906 of 28,621 (66%) *P. tecunumanii* transcripts. Coding regions were predicted for 14,423 *F. circinatum* transcripts using GeneMarkS-T and best-hit selection of BLASTp hits resulted in alignments for 12,985 (90%) proteins (Table S1). The majority of best hits (12,558) originated from *Fusarium* species. EggNOG annotation assigned 13,948 (97%) *F. circinatum* sequences to families and InterProScan annotation assigned domains to 9405 (65%) proteins, resulting in a total of 14,185 (98%) annotated sequences, of which 5368 (37%) were assigned gene ontology (GO) terms. Only 4516 (31%) *F. circinatum* sequences had putative alignments to PHI-base (Table S2). GhostKOALA assigned KO numbers to 27,973 (53%) *P. patula* transcripts, 12,038 (42%) *P. tecunumanii* transcripts and 4225 (29%) *F. circinatum* transcripts (Table S3).

### 2.2. Transcriptome Profiling

An average of 69.9 ± 6.8% *P. patula* reads mapped to the Pipt_v2.0 transcriptome and 71.3 ± 7.8% *P. tecunumanii* reads mapped to the Pnte_v1.0 transcriptome (Table S4). Additionally, for 3- and 7-dpi respectively, an average of 0.04 ± 0.01% and 0.05 ± 0.03% reads from mock-inoculated *P. patula* samples mapped to the *F. circinatum* reference transcriptome, with 0.08 ± 0.01% and 0.82 ± 0.42% reads mapped from inoculated samples. Comparably for *P. tecunumanii*, an average of 0.02 ± 0.01% and 0.30 ± 0.17% reads mapped to the *F. circinatum* transcriptome from mock-inoculated samples, with 0.16 ± 0.04% and 1.62 ± 0.81% reads mapped from inoculated samples.

Filtering of the expression data identified 25,000, 20,614 and 5,003 expressed genes for *P. patula, P. tecunumanii* and *F. circinatum*, respectively, across all samples. Subsequent filtering of *F. circinatum* expression resulted in 4,354 high-confidence expressed genes (Table 1, Table S5). Differential expression (DE) analysis of inoculated samples (*P. tecunumanii* versus *P. patula*) identified 132 and 470 significant *F. circinatum* differentially expressed genes (DEGs) for 3- and 7-dpi, respectively (Table 1, Table S5). *P. patula* DE analysis identified 323 and 7453 significant DEGs (inoculated versus mock-inoculated) at 3- and 7-dpi, while 735 and 2499 significant DEGs were identified for *P. tecunumanii* (Table 1, Tables S6 and S7).

### 2.3. Over-Represented GO Terms within Pathogen Datasets

GO enrichment analysis in the high confidence expressed *F. circinatum* genes showed shared biological process (BP), cellular compartment (CC) and molecular function (MF) terms between all data sets related to ribosomes, translation and lipid metabolism, indicative of growth, as well as BP terms related to responses to farnesol (Figure S3, Table S8). BP terms related to pectin hydrolysis were only enriched in the 3-dpi *P. patula* data set. Most enriched terms in the *P. tecunumanii* 3-dpi data set were also enriched in both the *P. tecunumanii* and *P. patula* 7-dpi data sets. This included CC terms related to membranes and mitochondria, MF terms related to hydrolysis, lipid binding and oxidoreductases, and BP terms related to responses to oxidative stress and respiration (Figure S3, Table S8). For both hosts at 7-dpi, there were also many enriched BP terms related to localization, cell wall organization, alcohol biosynthesis and sterol biosynthesis.

Further GO enrichment analysis of *F. circinatum* DEGs between *P. tecunumanii* and *P. patula* at both timepoints was performed to identify differences in pathogen responses between hosts (Table S9). Few terms were enriched for *F. circinatum* genes up-regulated during infection of *P. tecunumanii* relative to *P. patula*. No terms were enriched in the 3-dpi data set, while there was enrichment for the CC term, extracellular region, and the BP terms, carbohydrate metabolic process, and, oxidation-reduction process, in the 7-dpi data set. In contrast, many terms were enriched for *F. circinatum* genes down-regulated in inoculated *P. tecunumanii* relative to *P. patula* samples. At both time points, cytoplasmic translation terms were enriched in all three GO categories (Table S9). At 3-dpi, BP terms related to glycolysis and energy production were enriched, while at 7-dpi MF and BP terms related to sterol and alcohol biosynthesis were enriched. Due to the enrichment of terms related to ergosterol biosynthesis in the high confidence 7-dpi data set from both hosts, as well as in the down-regulated 7-dpi data set, this pathway was investigated further.

### 2.4. Transcriptional Responses Related to Ergosterol Biosynthesis in the Pathogen

**Figure 1:**
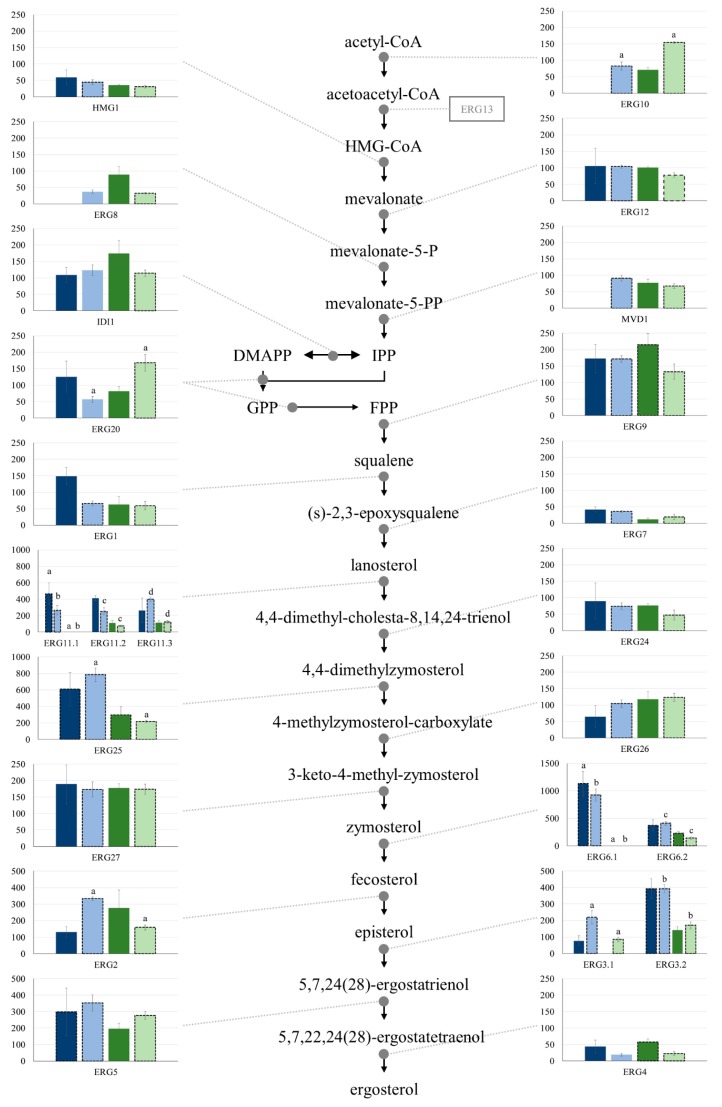
Expression of *Fusarium circinatum* ergosterol biosynthesis genes during infection. The y-axes represent average FPKM across biological replicates for inoculated samples from *P. patula* (blue) and *P. tecunumanii* (green) at 3- (dark colours) and 7- (light colours) dpi. Error bars represent the standard error of the mean (n = 3). Letters above bars indicate significant difference in expression (FDR < 0.05). Dashed black outlines indicate high-confidence expressed genes. ERG10 = acetyl-CoA acetyltransferase, HMG1 = 3-hydroxy-3-methylglutaryl-CoA (HMG-CoA) reductase, ERG12 = mevalonate kinase, ERG8 = phosphomevalonate kinase, MVD1 = mevalonate pyrophosphate decarboxylase, IDI1 = isopentenyl diphosphate:dimethylallyl diphosphate isomerase, ERG20 = geranylgeranyl diphosphate synthase, ERG9 = farnesyl-diphosphate farnesyltransferase, ERG1 = squalene epoxidase, ERG7 = lanosterol synthase, ERG11 = CYP51/sterol-14α-demethylase, ERG24 = δ14-sterol reductase, ERG25 = C-4 methylsterol oxidase, ERG26 = sterol-4α-carboxylate 3-hydrogenase, ERG27 = 3-keto steroid reductase, ERG6 = sterol 24-C-methyltransferase, ERG2 = C-8 sterol isomerase, ERG3 = δ7-sterol 5-desaturase, ERG5 = CYP61a/sterol 22-desaturase, ERG4 = δ24(24(1))-sterol reductase. ERG13 (HMG-CoA synthase) was absent from the transcriptome.

Candidate genes for all ergosterol biosynthesis steps could be identified in the *F. circinatum* transcriptome except *HMG-CoA synthase* (Table S10). Due to the physiological importance of *HMG-CoA synthase*, this likely indicates incompleteness of the genome rather than absence of the gene. *ERG10* and *ERG20* showed significantly lower expression during infection of *P. patula* than *P. tecunumanii* at 7-dpi (Figure 1). While this indicates that *F. circinatum* produces more farnesyl diphosphate (FDP) during infection of *P. tecunumanii*, FDP is the precursor for a wide array of metabolites. Conversely, five genes involved in late ergosterol biosynthesis, *CYP51/sterol-14α-demethylase* (*ERG11*), *C-4 methylsterol oxidase* (*ERG25*), *sterol 24-C-methyltransferase* (*ERG6*), *C-8 sterol isomerase* (*ERG2*) and *δ7-sterol 5-desaturase* (*ERG3*), were expressed at lower levels during infection of *P. tecunumanii* than *P. patula* at 7-dpi (Figure 1).

### 2.5. Over-Represented GO Terms within Host Datasets

Few GO terms were enriched for *P. patula* DEGs at 3-dpi (Table S11). Analysis of up-regulated DEGs identified the CC terms, cell wall and external encapsulating structure, as well as the BP terms, syncytium formation, cytoplasmic translation, response to oxygen-containing compound and response to chitin. All DEGs underlying the enriched syncytium formation term were predicted expansins, proteins involved in cell wall relaxation. The only enriched GO term in the down-regulated DEGs at 3-dpi was the MF term xyloglucan:xyloglucosyl transferase activity, an enzymatic activity involved in cell wall reinforcement.

For up-regulated DEGs at 7-dpi, most enriched CC terms were related to the nucleus, mitochondria, cytoplasm and ribosomes (Table S11). Enriched MF terms included many terms related to ribosomal activity and control of reactive oxygen species (ROS) during a defence response. Many BP terms were enriched, including a wide array of known defence related terms. Among these were terms related to: apoptosis, ROS production, response to oxidative stress, increased cytokine production, terpenoid biosynthesis, the lipoxygenase pathway, response to chitin (similar to the 3-dpi data set), MAPK signaling, responses to salicylic acid (SA), jasmonic acid (JA) and ethylene (ET), as well as JA-mediated induced systemic resistance (ISR) signaling. All BP terms enriched at 3-dpi were also enriched at 7-dpi except syncytium formation, however, a larger complement of expansins were up-regulated at 7- than 3-dpi. For down-regulated genes, the enriched CC terms were all plastid related and the only enriched MF term was beta-amylase activity. The enriched BP terms included plastid organization and plastid processes, such as starch metabolism, apoptosis and hypersensitive response (HR), as well as SA responses and SAR.

For *P. tecunumanii* up-regulated DEGs, the enriched CC terms shared between timepoints were mostly related to the cytoskeleton, coated vesicles, vacuoles and the proteasome (Table S12). Most enriched MF terms shared between timepoints, as well as the terms unique to 3-dpi, were related to protein and nucleic acid binding (Table S12). The MF terms specific to the 7-dpi up-regulated DEGs were predominantly related to hydrolases, including chitinase activity, and lipases. The 7-dpi unique MF terms contained the term, xyloglucan:xyloglucosyl transferase activity, enriched in the 3-dpi down-regulated *P. patula* dataset.

A large number of BP terms were enriched in the *P. tecunumanii* up-regulated DEGs at both time points (Table S12). Potential defence related BP terms enriched at both timepoints were related to response to chitin, ubiquitin-mediated proteolysis (UMP) and vesicle mediated transport. Enriched terms unique to the 3-dpi dataset were related to UMP, ET signaling, JA mediated ISR and terpenoid biosynthesis. The enriched terms unique to 7-dpi were related to the SA response, SA-mediated SAR, JA responses, ET biosynthesis and the biosynthesis of various phytoalexins, including camalexin.

Enriched GO terms for down-regulated *P. tecunumanii* DEGs at both timepoints were mainly related to cellular growth and replication (Table S12). Most enriched BP terms for down-regulated DEGs at 3-dpi were also enriched for down-regulated DEGs at 7-dpi.

### 2.6. Transcriptional Responses Related to Host Phytohormone Signalling

**Figure 2:**
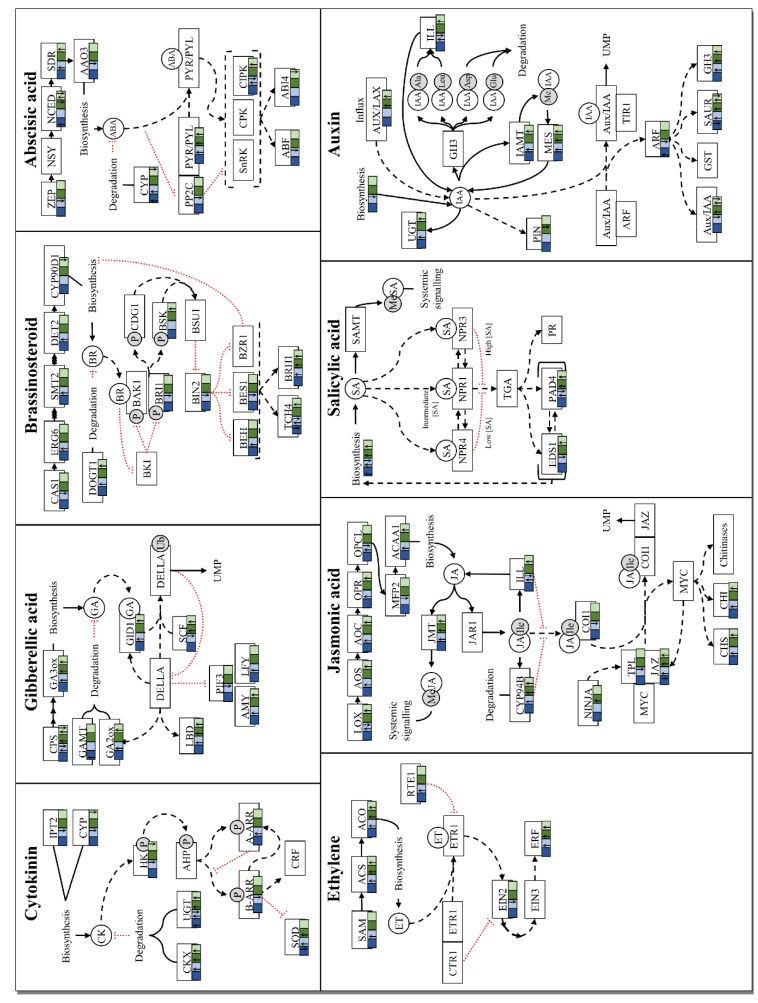
Summary of phytohormone related host DEGs during *F. circinatum* challenge. Up- and down-regulation (inoculated relative to mock-inoculated) of genes related to cytokinin, abscisic acid, gibberellic acid, brassinosteroid, ethylene, jasmonic acid, salicylic acid and auxin signalling, in *P. patula* (blue) and *P. tecunumanii* (green) at three (dark colours) and seven (light colours) days post inoculation, are indicated by arrows (**↑** and **↓** respectively; Additional file 5: Tables S4 and S5). Dotted red lines indicate suppression, dashed black lines indicate positive interaction, solid black lines indicate enzymatic reactions. Borderless text indicates processes, square bordered text indicates proteins, round bordered text indicates compounds. AAO3—abscisic-aldehyde oxidase; A-ARR—type A Arabidopsis response regulator; ABA—abscisic acid; ABF—ABA response factor; ABI—ABA insensitive; ACAA1—acetyl-Coenzyme A acyltransferase 1; ACC—1-aminocyclopropane-1-carboxylic acid; ACO—ACC oxidase; ACS—ACC 1-aminocyclopropane-1-carboxylic acid synthase; AHP—Arabidopsis histidine phosphotransferase; Ala—alanine; AMY—α-amylases; AOC—allene oxide cyclase; AOS—allene oxide synthase; ARF—auxin response factor; Asp—aspartic acid; Aux/IAA—auxin inhibitor; AUX/LAX—auxin influx carriers; BAK1—BRI1-associated kinase; B-ARR—type B Arabidopsis response regulator; BIN2—BR insensitive 2; BR—brassinosteroid; BRH1—brassinosteroid-responsive RING H2; BRI1—brassinosteroid insensitive 1; BSK—BR-signalling kinase; BSU1—protein phosphatase BRI1 suppressor; CAS1—cycloartenol synthase; CDG1—constitutive differential growth 1; CHI—chalcone-flavone isomerase; CHS—chalcone synthase; CIPK—Cbl-interacting protein kinase; CK—cytokinin; CKX—CK dehydrogenase; COI1—coronatine insensitive 1; CPS—ent-copalyl diphosphate synthase; CRF—CK response factor; CTR1—Raf-like ser/thr kinase; CYP—Cytochrome P450 family protein; DOGT1—DON-glucosyltransferase 1; EDS1—enhanced disease susceptibility 1; EIN—ET insensitive; ERF—ET response factor; ERG6—sterol 24-C-methyltransferase; ET—ethylene; ETR1—ET receptor; GA—gibberellic acid; GA2ox—GA 2-oxidase; GA3ox—GA 3-oxidase; GAMT—GA methyl transferase; GH3—Gretchen Hagen3 family protein; GID1—GA insensitive dwarf 1; Glu—glutamine; GST—glutathione-S-transferase; HK—histidine kinase; IAA—indole-3-acetic acid; IAMT—IAA methyl transferase; ILL—JA-Ile/IAA-amino acid hydrolase; IPT2—isopentenyl transferase; JA—jasmonic acid; JA-Ile—jasmonoyl-isoleucine; JAR1—JA-amino acid synthetase; JAZ—jasmonate zim-domain family transcription repressor proteins; JMT—JA methyl transferase; LBD—lob domain-containing protein; Leu—leucine; LFY—LEAFY; LOX—lipoxygenase; MeJA—methyl-jasmonate; MES—methyl esterase; MeSA—methyl-salicylate; MFP2—multifunctional protein 2; MYC—JA responsive transcriptome factor; NCED—9-cis-epoxycarotenoid dioxygenase; NINJA—novel interactor of JAZ; NPR—non-expressor of PR; NSY—neoxanthin synthase; OPCL—OPC8-CoA ligase; OPR—12-oxophytodienoate reductase; PAD4—phytoalexin deficient 4; PIF3—phytochrome-interacting factor 3; PIN—auxin efflux transporter; PP2C—type 2C protein phosphatases; PR—pathogenesis related proteins; PYR/PYL—ABA receptors; RTE1—reversion-to-ethylene sensitivity 1; SA—salicylic acid; SAM—S-adenosyl-methionine synthetase; SAMT—SA methyl transferase; SAUR—small auxin up RNA protein; SCF—Skp-cullin-F-box complex; SDR—xanthoxin dehydrogenase; SMT2—24-methylenesterol C-methyltransferase; SOD—superoxide dismutase; TCH4—Xyloglucan endotransglucosylase hydrolase protein; TGA—TGA family transcription factors; TPL—topless; Ub—ubiquitin; UGT—UDP-Glycosyl/Glucosyl/Glucuronosyl transferase; UMP—ubiquitin mediated proteolysis; ZEP—zeaxanthin epoxidase.

Phytohormones play crucial roles during growth and development. Interactions between these hormones also serve to regulate gene expression during stress responses. Hormone biosynthesis and signaling related DEGs were investigated to identify putative pathways involved in the pine-*Fusarium* interaction due to the enrichment of different phytohormone related GO terms in the up-regulated data at 3- and 7-dpi in *P. tecunumanii*, as well as in both the up- and down-regulated data at 7-dpi in *P. patula* (Figure 2, Tables S13 and S14).

#### 2.6.1. Cytokinin

At both time points in *P. tecunumanii*, there was up-regulation of UDP-glycosyl transferase (*UGT*) and cytokinin oxidase/dehydrogenase (*CKX*) genes, enzymes related to cytokinin (CK) degradation, with more *UGTs* up-regulated at 7-dpi (Figure 2, Table S14). There was also down-regulation of cytochrome P450s (*CYP*) related to CK biosynthesis and histidine kinase (*HK*) receptor genes at 7-dpi. Increased degradation and down-regulation of biosynthesis indicate suppression of CK at both time points. In *P. patula*, the only CK related DEGs at 3-dpi were up-regulated *UGT* genes, while at 7-dpi, similar to *P. tecunumanii*, there was up-regulation of *UGT* and *CKX* genes as well as down-regulation of *HK* genes (Figure 2, Table S13). Furthermore, two *A-ARR* genes, negative regulators of CK signaling, showed up-regulation and two *B-ARR* genes, positive regulators of CK signaling and *A-ARRs*, showed down-regulation in *P. patula* at 7-dpi. This, together with the up-regulation of superoxide dismutase (*SOD*) genes, indicate suppression of CK signaling.

#### 2.6.2. Gibberellic Acid

At 3-dpi in *P. patula*, the only gibberellic acid (GA) related DEG was an up-regulated GA methyl transferase (*GAMT*, Figure 2, Table S13). Unlike other phytohormones, methylation results in GA degradation (Eckardt, 2007). GA 3-oxidase (*GA3ox*) and putative ent-copalyl diphosphate synthase (*CPS*) genes were up-regulated at both time-points in *P. tecunumanii*, as well as at 7-dpi in *P. patula* indicating possible GA biosynthesis at 7-dpi in both hosts. However, there were also down-regulated *CPS* genes, with more *CPS* genes down-regulated in *P. patula* than *P. tecunumanii*. *GID1*, *GA2ox*, *LBD* and *AMY* genes were up-regulated in both hosts at 7-dpi. A *LFY* gene was up-regulated at 7-dpi in *P. patula* and phytochrome-interacting factor 3 (*PIF3*) genes were down. While up-regulation of *LFY* and *AMY* genes indicate the presence of GA signaling between 3 and 7-dpi, up-regulation of DELLA responsive *GA2ox*, *GID1* and *LBD* indicate suppression of GA signaling at 7-dpi in both hosts.

#### 2.6.3. Brassinosteroids

A steroid biosynthesis gene was down-regulated at 7-dpi in *P. tecunumanii*, while brassinosteroid (BR) signalling kinase 1 (*BSK1*) was up-regulated (Figure 2, Table S14). At both time points in *P. tecunumanii*, there was also up-regulation of putative brassinosteroid-responsive RING H2 (*BRH1*) genes. In *P. patula*, some steroid biosynthesis and brassinazole resistant (*BZR*) genes, as well as *BRH1*, were up-regulated at 7-dpi; cycloartenol synthase (*CAS1*), brassinosteroid-insensitive 1 (*BRI1*), *BIM1* (a BZR2 synergist) and brassinosteroid-insensitive 2 (*BIN2*) genes, however, were down. BR signaling has been shown to rapidly decrease the expression of *BRH1*, while the pathogen elicitor chitin increases expression [29]. Thus, BR signaling appeared to be absent at 3-dpi in both hosts and up-regulation of BRH1 at 7-dpi was indicative of BR signaling suppression. DON-glucosyltransferase 1 (*DOGT1*) genes, associated with inactivation of BRs and CKs through glucosylation [30,31], were up-regulated at 7-dpi in both species, further indicating suppression of both CK and BR signaling.

#### 2.6.4. Abscisic Acid

In *P. tecunumanii* a 9-*cis*-epoxycarotenoid dioxygenase (*NCED*) was down-regulated at both time points, however, at 7-dpi another *NCED*, as well as a putative xanthoxin dehydrogenase (*SDR*) was up-regulated and an abscisic acid (ABA) hydroxylase was down-regulated (Figure 2, Table S14). This could indicate suppression of ABA biosynthesis at 3-dpi and induction at 7-dpi in the resistant host. In *P. patula*, an *NCED* and an ABA hydroxylase were up-regulated at 3-dpi. At 7-dpi, although an *SDR1* was up-regulated, other ABA biosynthesis genes, zeaxanthin epoxidase (*ZEP*) and abscisic-aldehyde oxidase (*AAO3*), were down-regulated while an ABA hydroxylase and a carotenoid cleavage dioxygenase 8 (*CCD8*) were up-regulated. *CCD8* diverts carotenoid metabolism away from Zeaxanthin. This indicates suppression of ABA biosynthesis and increased degradation at 7-dpi in the susceptible host.

ABA receptors were up-regulated at both time points in *P. tecunumanii* and at 7-dpi in *P. patula*. A type 2C protein phosphatase (*PP2C*) gene was down-regulated at 7-dpi in *P. patula*. The up-regulation of receptors and a *CIPK20* could indicate ABA signaling at both time points in *P. tecunumanii*. At 7-dpi in *P. patula*, down-regulation of ABA biosynthesis indicates suppression of ABA levels, while up-regulation of receptor and *CIPK20* genes with down-regulation of a *PP2C* suggest ABA signaling.

Cross talk between CK and ABA signaling is mediated by ABA-insensitive 4 (ABI4) and ABI5, positive regulators of ABA signaling, and A-ARRs. ABI5 activity is attenuated by interaction with A-ARRs, allowing A-ARRs to negatively regulate both CK and ABA signaling [32]. ABI4 positively regulates A-ARR5, resulting in suppression of CK responses by ABA signaling [33]. An ABI4 gene was down-regulated at 7-dpi in *P. tecunumanii*, which could indicate a lack of ABA signaling despite the up-regulation of receptors. As ABA has been implicated in suppression of GA responses by stabilizing DELLA proteins, preventing their degradation [34], the up-regulation of ABA biosynthesis in *P. tecunumanii* could be related to GA suppression.

#### 2.6.5. Ethylene

1-aminocyclopropane-1-carboxylic acid (ACC) synthase (*ACS*), ACC oxidase (*ACO*) and a large amount of ET response factor (*ERF*) genes were up-regulated at both time points in *P. tecunumanii* and at 7-dpi in *P. patula* (Figure 2, Table S13). More *ERFs* were up-regulated at 3-dpi in *P. tecunumanii* than at 7-dpi. There was also a S-adenosyl-methionine (*SAM*) synthetase up- and an ET insensitive 2 (*EIN2*) gene down-regulated at 7-dpi in *P. patula*. The up-regulation of ET biosynthesis genes and *ERFs* indicates active ET signaling at both timepoints in *P. tecunumanii* and at 7-dpi in *P. patula*. However, down-regulation of *EIN2* and up-regulation of a predicted reversion-to-ethylene sensitivity 1 (*RTE1*), a known negative regulator of ET signaling [35], in *P. patula* could interfere with ET signaling. ET signaling has been implicated in the suppression of ABA biosynthesis as well as negative regulation of ABA signaling [36]. Thus, despite up-regulation of ABA receptors at both time points in *P. tecunumanii*, up-regulation of ET biosynthesis could indicate suppression of ABA signaling by ET.

#### 2.6.6. Jasmonic Acid

At 3-dpi in *P. patula*, a single jasmonate zim-domain (*JAZ*) family transcription repressor was down-regulated (Figure 2, Table S13). At 7-dpi in both species lipoxygenase 5 (*LOX5*), allene oxide synthase (*AOS*), allene oxide cyclase 3 (*AOC3*), 12-oxophytodienoate reductase 3 (*OPR3*), JA-Ile/IAA-amino acid hydrolases (*ILL*) and chalcone synthase (*CHS*) genes were up-regulated. Additionally, *LOX1* and *OPR2* were up-regulated in *P. patula*. JA-Ile 12-hydroxylases (*CYP94B*), *JAZ* and jasmonate methyl transferase (*JMT*) genes were up-regulated at both time points in *P. tecunumanii* and at 7-dpi in *P. patula*. Thus, few JA related genes were DE at 3-dpi in both species while at 7-dpi there was increased JA biosynthesis and responses. Although this indicates a role for JA at 7-dpi in both species, there was down-regulation of a coronatine insensitive 1 (*COI1*) gene at 7-dpi in *P. patula*, which could suppress JA responses.

#### 2.6.7. Salicylic Acid

There are two main routes for SA production in plants, the isochorismate (IC) and phenylalanine ammonia-lyase (PAL) pathways, both originating from the shikimate pathway product chorismate. In *P. tecunumanii*, shikimate pathway and PAL genes were up-regulated at both time points, with more genes up-regulated at 7-dpi (Figure 2, Table S14). Phytoalexin deficient 4 (*PAD4*) was also up-regulated at both time points, however, enhanced disease susceptibility 1 (*EDS1*) was only up-regulated at 7-dpi. Conversely, in *P. patula*, while shikimate pathway and PAL genes were also up-regulated at 7-dpi, *EDS1* and the majority of *PAD4* DEGs were down-regulated. Isochorismate synthase (*ICS*), the first step in the IC pathway [37], was also down-regulated at 7-dpi. Furthermore, isochorismatase, an enzyme that diverts isochorismate away from SA synthesis, was up-regulated. PR-1 genes, classic SA response genes [38], were up-regulated at both time points in *P. tecunumanii*, while in *P. patula*, they were up-regulated at 7-dpi and down-regulated at 3-dpi (Tables S13 and S14). Thus, while SA signaling appeared to be active at 7-dpi in *P. tecunumanii*, at 7-dpi in *P. patula* there were indications of SA biosynthesis and signaling suppression.

#### 2.6.8. Auxin

In *P. patula*, the only auxin related DEGs at 3-dpi were an up-regulated auxin response factor (*ARF*) 19 and a down-regulated small auxin up-regulated (*SAUR*)-like gene (Figure 2, Table S13). At 7dpi, an auxin biosynthesis gene (*YUCCA*) was down-regulated in *P. patula*. As *YUCCA* is only involved in one auxin biosynthesis pathway, auxin could still be produced via other routes [39]. *ARF4*, *ARF6*, *ILL5* (IAA-amino acid hydrolase), *CAND1* (Cullin-associated and neddylation dissociated 1) and three *Aux/IAA* auxin repressor genes were also down-regulated at 7-dpi in *P. patula*, indicating suppression of auxin signaling, despite the up-regulation of putative *SAUR* genes as well as *GH3* (Gretchen Hagen 3 acyl acid amido synthetase family proteins), *IAMT1* (IAA carboxyl methyltransferase 1) and *ILL6* genes. In *P. tecunumanii*, no auxin biosynthesis genes were differentially expressed, however, auxin influx carrier genes were up-regulated at both time points and an auxin efflux carrier gene was up-regulated at 3-dpi. Furthermore, *Aux/IAA*, *GH3* and *SAUR* genes were up-regulated at both time points and there was up-regulation of *IAMT*, *ILL6* and *CAND1* genes as well as down-regulation of some *Aux/IAA* and *SAUR* genes at 7-dpi. The up-regulation of *ILL6* and influx proteins indicate an increase of auxin levels in *P. tecunumanii* at both time points, however, the efflux carrier indicates lower auxin levels at 3- than 7-dpi, which is reflected in the amount of auxin response DEGs.

A *MES1* gene, a methyl esterase capable of hydrolyzing MeSA, MeJA and MeIAA, was up-regulated in both hosts at 7-dpi. There was also up-regulation of MES17, a MeIAA specific methyl esterase, at 7-dpi in *P. tecunumanii*. Systemic signaling through hormone methyl esters requires demethylation to activate the hormone [40,41,42]. Thus, up-regulation of *IAMT1* and *MES* genes could indicate systemic signaling at 7-dpi in both hosts.

At 7-dpi, cullin genes were up-regulated in *P. tecunumanii* but down-regulated in *P. patula*. Cullins are critical structural proteins of Skp-Cullin-F-box (SCF) complexes. This could indicate a decreased ability to activate defence signaling in *P. patula* as SCF complex catalyzed ubiquitination is an important component of GA, JA and auxin signaling [43,44,45].

## 3. Discussion

The disparate *F. circinatum* resistance phenotypes of *P. patula* and *P. tecunumanii* [15] provided a pathosystem to study resistant and susceptible host-pathogen interactions between *Pinus* spp. and *F. circinatum*. The susceptibility of *P. patula* and resistance of *P. tecunumanii* to *F. circinatum* challenge was previously confirmed by the significant difference in lesion development between species, as well as mortality of *P. patula* seedlings, while *P. tecunumanii* seedlings showed signs of recovery [28]. Significantly higher read mapping to the *F. circinatum* transcriptome from inoculated relative to mock-inoculated samples, and up-regulation of most genes in the *F. circinatum* high confidence expressed genes, for each sample set supported the presence of pathogen sequence reads, as expected from the reported infection [28]. This was corroborated by higher mapping from 7- relative to 3-dpi inoculated samples, which is indicative of fungal growth.

Expression of *F. circinatum* genes indicated compromised ergosterol biosynthesis during infection of the resistant host at 7-dpi, which could increase pathogen susceptibility to PR-1 proteins [14]. All detected *F. circinatum* orthologs of five ergosterol biosynthesis genes, *ERG11*, *ERG25*, *ERG6*, *ERG2* and *ERG3*, were expressed at lower levels during infection of *P. tecunumanii* relative to *P. patula*. Azole group fungicides inhibit fungal growth by inhibiting ERG11, blocking ergosterol biosynthesis [46]. One known mechanism of azole resistance in *Candida albicans* results from a loss of function mutation of *ERG3*, without affecting virulence [47]. Conversely, in *F. graminearum ERG3* mutation has been associated with decreased virulence [48]. Although many fungal pathogens exhibit resistance to azole fungicides, plant coumarins have also been associated with ERG11 inhibition in *C. albicans* [49], and the transformation of *Arabidopsis* and barley with an *ERG11* targeting double-stranded RNA, to elicit host-induced gene silencing, resulted in complete immunity to *F. graminearum* [50]. Additionally, a tomato glycoalkaloid has been associated with suppression of ergosterol biosynthesis in *Saccharomyces cerevisiae* by inhibiting ERG6 [51] and treatment of *C. albicans* with the terpenoid farnesol resulted in down-regulation of *ERG11*, *ERG25*, *ERG6* and *ERG3* [52]. Thus, the lower relative expression of these genes during infection of the resistant host could aid in host resistance.

Enriched GO terms indicated a delayed and imprecise response to *F. circinatum* challenge by *P. patula*. The overrepresented GO terms for *P. tecunumanii* DEGs suggested active host defence responses at both time points, while defence related GO terms were only enriched at 7-dpi for *P. patula*. Additionally, in *P. tecunumanii* there was enrichment of ubiquitin-mediated proteolysis and cell cycle regulation terms in the up-regulated DEGs and DNA and cellular replication terms in the down-regulated data which were absent for *P. patula*. Ubiquitin-mediated proteolysis is a critical process for the activation and regulation of GA, JA, SA and auxin signaling pathways [43,44,45,53]. Several lines of evidence suggest the existence of a growth-defence trade-off in plants [54], thus decreasing replication could assist the host in mounting a successful defence. Furthermore, although ET, JA and SA related terms were enriched for the up-regulated 7-dpi *P. patula* DEGs, similar to *P. tecunumanii*, there was also enrichment for terms related to oxidative stress, apoptosis and ROS production. In plants, these responses are associated with HR [55], which has been linked to increased susceptibility against necrotrophic pathogens and has been shown to be promoted by necrotrophs to facilitate infection [56].

Phytohormone related DEGs at 3-dpi indicated roles for auxin and ET in *P. tecunumanii* defence responses. Plant defence against necrotrophic pathogens is usually associated with active signaling by both the ET and JA pathways [57,58,59,60]. However, while no JA biosynthesis genes were up-regulated, putative *JAZ* genes (repressors of JA responses) and a JA hydroxylase (involved in JA degradation) were up-regulated, indicating suppression of JA signaling. The jasmonate-insensitive 1 (JIN1, a.k.a. AtMYC2) MYC protein has been shown to negatively regulate *EIN3* expression, inhibiting the expression of *ERF1* [61]. Thus, JA suppression could allow for the activation of a larger repertoire of ET responses. Auxin has also been shown to have an antagonistic effect on JA signaling by stimulating the expression of JAZ proteins [62].

At 7-dpi, *P. tecunumanii* DEGs suggested the inclusion of JA and SA in host resistance. JA biosynthesis genes were up-regulated, indicative of JA signaling and, while more ET biosynthesis genes were up-regulated, markedly fewer *ERFs* were up-regulated, reflecting the expected JA/ET antagonism [61]. Additionally, a larger array of auxin response genes were differentially regulated relative to 3-dpi. A synergistic interaction between auxin and JA signaling has been associated with enhanced host resistance to necrotrophic pathogens [45,63]. There was also up-regulation of SA biosynthesis genes and the SA response marker genes *PAD4* and *EDS1* [64,65], as well as more *PR-1* genes at 7- relative to 3-dpi. Therefore, while at 3-dpi *P. tecunumanii* seemed to induce ET and auxin while suppressing JA signaling, at 7-dpi, transcriptomic responses indicated a complex regulation of host defence using auxin, ET, JA and SA. Although SA signaling is usually classified as antagonistic to both auxin and JA signaling, synergistic interactions also exist [32,45,66]. SA signaling has been shown to induce JA biosynthesis and modulate JA defences in *Arabidopsis* effector-triggered immunity [67,68]. The early auxin responsive GH3 proteins play an important role in mediating crosstalk between auxin, JA and SA [45]. The core JA signaling component JAR1, which is required for production of the bioactive JA-Ile conjugate, is a GH3 [69], and increased *GH3* expression has been shown to simultaneously induce the SA pathway and derepress the auxin pathway [70]. The SA conjugate salicyloyl-aspartate has also been implicated as a signaling molecule to induce systemic resistance [70,71]. Thus, auxin signaling could play an important role in coordinating and integrating phytohormone defence pathways, similar to the central role played in growth and development [45,72,73,74].

Host responses at 3-dpi in *P. patula* indicated a lack of defence and increased membrane permeability. Less than 400 genes were differentially expressed at 3-dpi in the susceptible host. A previous study investigating *P. patula* responses to *F. circinatum* challenge at 1-dpi identified even fewer DEGs [75]. Although overrepresentation of the GO term, response to chitin, at 3-dpi indicated fungal perception [76,77], there was no enrichment for defence related terms and few phytohormone related or PR family DEGs. Furthermore, there was overrepresentation of the GO term, syncytium formation, in the up-regulated DEGs and the term, xyloglucan:xyloglucosyl transferase activity, in the down-regulated DEGs. Expansins are involved in loosening of the cell wall associated with growth as well as symbiotic interactions [78,79]. Suppression of these proteins has been associated with increased resistance to necrotrophic pathogens [79,80]. Xyloglucan:xyloglucosyl transferases are involved in covalent cross-linking of cell wall polymers, such as xylose, and have been associated with cell wall reinforcement through xyloglucan remodeling [81,82]. Increased levels of cell-wall bound xylose have been associated with resistance to necrotrophic pathogens, while decreased levels have been associated with susceptibility, in *Arabidopsis* [83]. Thus, the up-regulation of expansins and the down-regulation of xyloglucan:xyloglucosyl transferases could contribute to susceptibility in *P. patula* by increasing membrane permeability. Combined with the seeming lack of defence responses, this could indicate effector triggered susceptibility at 3-dpi.

Despite the enrichment of defence related GO terms, *P. patula* DEGs at 7-dpi suggested an impaired phytohormone defence response against *F. circinatum*. Regarding SA related DEGs, the biosynthesis gene *ICS2*, as well as the SA response marker genes *EDS1* and *PAD4* were down-regulated. Thus, unlike *P. tecunumanii*, the SA defence pathway appeared to be suppressed at 7-dpi. Biosynthesis genes for ET and JA, as well as many *ERF* and *JAZ* genes, were up-regulated. Auxin responsive *SAUR* and *GH3* genes were up-regulated, while *Aux/IAA* and *ARF* genes were down-regulated. Although this could indicate signaling by these phytohormones, key JA and ET signaling genes, *COI1*, *TPL* and *EIN2*, were down-regulated and a negative regulator of ET signaling, *RTE1*, was up-regulated, indicating that these signaling pathways could be compromised. Auxin signaling has been shown to have an antagonistic effect on JA signaling by stimulating the expression of JAZ proteins [62,84,85] as well as inducing the expression of ERFs [86,87,88]. However, similar to JA and GA signaling, auxin signaling requires SCF-mediated repressor degradation [44,89]. Down-regulation of *CAND1* and *cullin* genes suggests a decrease in available SCF complexes. Thus, despite the difference in the number of DEGs between time points, indicating a delayed response, immune signaling appeared to be compromised at 7-dpi. Nonetheless, the large number of up-regulated defence-related and *PR*-genes suggest some form of defence signaling. One of the down-regulated auxin response factors, ARF2, has been implicated in the negative regulation of COI1-independent defence responses in *A. thaliana* [90]. Similarly, the up-regulation of a rice *GH3* has been associated with SA- and JA-independent immunity in rice [80]. Consequently, the differential regulation of defence related genes at 7-dpi could be the result of phytohormone-independent signaling.

In summary, this dual RNA-seq study suggested that ergosterol could be required for *F. circinatum* virulence in pine and identified phytohormone signaling pathways potentially involved in host resistance and susceptibility. This study purports that the observed reduction of *F. circinatum* ergosterol biosynthetic gene expression could compromise this pathway, combined with the up-regulation of host PR-1 genes, this could be a key factor contributing to host resistance. Future work to determine the effect of supressing *F. circinatum* ergosterol biosynthesis on host susceptibility and pathogen response to host metabolite treatment is required to provide further support for the observed responses. Furthermore, DEGs in *P. tecunumanii* indicated the integration and coordination of auxin, ET, JA and SA mediated defence responses could be required for resistance, while the absence of defence responses at 3-dpi and the down-regulation of phytohormone signaling components at 7-dpi in *P. patula* suggested pathogen inhibition of host responses. To the authors’ knowledge, these results represent the first comprehensive investigation of *F. circinatum* gene expression during pathogenesis of pine, as well as the first comparison of host responses to *F. circinatum* between two different pine species. Although an ideal comparison would have been to compare responses between resistant and susceptible genotypes of each species, no susceptible *P. tecunumanii* LE genotypes are known and even the most tolerant *P. patula* genotypes are still susceptible. Despite this limitiation, the current approach improves knowledge on the pine-*F. circinatum* host pathogen interaction, as well as adding to the limited knowledge of defence responses in conifers. While this study investigated how these resistant and susceptible hosts respond to *F. circinatum* challenge on a transcriptomic level, under the assumption that changes in expression are implicated in defence, it is possible that differences in basal defences between the species would contribute to the resistant or susceptible outcome. Future work is required to investigate the role of physiological differences between these hosts.

## 4. Materials and Methods

### 4.1. Read Data From F. circinatum Inoculation Trial

RNA-sequencing libraries for *P. patula* and *P. tecunumanii*, generated from a *F. circinatum* inoculation trial, were obtained from the National Center for Biotechnology Information (NCBI) Sequence Read Archive (SRA). In brief, six-month-old seedlings, from open-pollinated families, were inoculated with *F. circinatum* isolate FCC3579 and mock-inoculated using sterile glycerol [28]. The top 1 cm of shoot tissue was harvested at 3- and 7-days post inoculation (dpi) for three biological replicates per treatment group. A biological replicate consisted of tissue pooled from 16 seedlings. The complete disease progression was reported previously [28]. At 3- and 7-dpi, disease symptoms were not observed on either host species, however, at 14-dpi *P. patula* showed marked lesions from the point of inoculation. The expected difference in host resistance, represented by the difference in lesion development rates [15,28], was clearly visible by 21-dpi, with pronounced lesion development on inoculated *P. patula* and only mild discolouration on inoculated *P. tecunumanii* (Figure S1). RNA was extracted using the Plant/Fungi RNA Purification Kit (Norgen Biotek Corp., Thorold, ON, Canada) and sent to Novogene (Novogene Corporation Inc, Chula Vista, CA, USA) for strand specific RNA-sequencing on an Illumina HiSeq2500 (Illumina, San Diego, CA, USA), PE125 for 3-dpi samples and PE150 for 7-dpi samples.

### 4.2. Reference Sequences

Host reference transcriptomes, Pipt_v2.0 for *P. patula* and Pnte_v1.0 for *P. tecunumanii*, were obtained from the NCBI Transcriptome Shotgun Assembly (TSA) database. Predicted proteins were assigned to MapMan functional categories using Mercator [91] with default parameters and inclusion of all available databases. The *F. circinatum* reference transcriptome was obtained by extracting the longest transcript sequence for all predicted genes (15,049) from the *F. circinatum* (strain FSP34) genome sequence [92]. The extracted transcript sequences were annotated with EnTAP debug_0.7.4.6 [93]. Open reading frame prediction was performed using GeneMarkS-T v5.1 March 2014 [94] followed by BLASTp similarity searches using the NBCI’s non-redundant protein database (release-84), RefSeq complete protein database (release-84) and the UniProtKB/Swissprot database (release-2017_09) through diamond 0.9.9 [95] (minimum-query-coverage = 80%, minimum target coverage = 60%, minimum *e*-value = 1 × 10^−5^), as well as EggNOG 0.99.1 [96] and InterProScan 5.25-64.0 [97] for orthologous group and GO term assignment (Figure S2). Predicted *F. circinatum* proteins were also aligned to the pathogen-host interactions (PHI) database 4.2 [98] to identify potential pathogenicity and virulence factors using diamond. Kyoto Encyclopaedia of Genes and Genomes (KEGG) orthology (KO) annotation was performed on all transcriptomes using GhostKOALA [99]. Read data and reference transcriptomes for *P. patula* (BioProject PRNJA416698) and *P. tecunumanii* (BioProject PRJNA416697) supporting the results of this article are available through the NCBI.

### 4.3. Mapping and Gene Expression Analysis

For both host species, the host and pathogen reference transcriptomes were combined (Figure S2). Read mapping and expression quantification, against the combined references, was performed using Kallisto 0.42.4 [100], with sequence bias correction and 40 bootstrap samples. The count data was imported into R 3.4.2 [101], using tximport 1.4.0 [102], for differential expression (DE) analysis. Fungal genes expressed at equal levels in inoculated and mock-inoculated samples, when normalizing against the full read set, were likely due to endophyte expression. Therefore, a high-confidence pathogen expressed gene set was produced to exclude potential endophyte contamination by performing a DE analysis using the full count data set (including both host and pathogen mapped reads) for each host (Figure S2). *F. circinatum* genes significantly up-regulated in inoculated relative to mock-inoculated samples were considered high confidence expressed genes for each time point. Significantly down-regulated *F. circinatum* genes, in inoculated relative to mock-inoculated samples, could represent potential endophyte genes and were discarded for downstream analysis. For pathogen DE analysis, *F. circinatum* count data from both host species was combined. Pathogen expression data was removed from the count data for host DE analysis. Transcripts with less than 20 reads from at least three read libraries were classified as low expression transcripts and filtered out. DE testing was performed with DESeq2 1.18.1 [103] using a Wald test with Benjamini and Hochberg (BH) false discovery rate (FDR) correction (*p* < 0.05, Abs|Log2(Fold-change)| ≥ 0.5). Biological pathways related to the differentially expressed genes (DEGs) were investigated using Mercator annotations with MapMan v3.5.1R2 [104], as well as KEGG orthology with KEGG mapper reconstruct pathway tool [105,106,107].

### 4.4. Gene Ontology Enrichment Analysis

DEGs for each comparison were divided into up- and down-regulated subsets (inoculated versus mock-inoculated for host transcripts; *P. tecunumanii* vs. *P. patula* for *F. circinatum* transcripts, Figure S2). Significant enrichment of GO terms (BH FDR, *p* < 0.10), relative to the transcriptome annotation for each species, was determined for each high confidence pathogen expressed gene set as well as the host and pathogen DEG subsets using GOSeq 1.28.0 [108].

## Figures and Tables

**Table 1:** Summary of significant differentially expressed genes identified for each comparison.

Category	Genes Up-Regulated ^a^	Genes Down-Regulated ^a^
Differentially expressed host genes ^b^		
*P. patula* 3-dpi	209	114
*P. patula* 7-dpi	4116	3337
*P. tecunumanii* 3-dpi	625	110
*P. tecunumanii* 7-dpi	1987	512
*F. circinatum* high confidence expressed genes ^c^		
3-dpi *P. patula* samples	210	0
7-dpi *P. patula* samples	2372	5
3-dpi *P. tecunumanii* samples	1409	0
7-dpi *P. tecunumanii* samples	4125	1
Differentially expressed *F. circinatum* genes ^d^		
3-dpi inoculated samples	39	93
7-dpi inoculated samples	264	206

^a^ Significant (FDR < 0.05), up- (log2(Fold Change) > 0.5) and down-regulated (log2(Fold Change) < −0.5), differentially expressed genes identified using the Wald test (Benjamini & Hochberg FDR correction) with DESeq2. ^b^ Host genes differentially expressed in inoculated relative to mock-inoculated host expression data. ^c^ *F. circinatum* genes differentially expressed in inoculated relative to mock-inoculated samples in the full expression data set (including both host and pathogen mapped reads) for each host. Up-regulated genes represent high confidence *F. circinatum* expressed genes. Down-regulated genes were excluded from downstream analysis. ^d^ *F. circinatum* genes differentially expressed in *P. tecunumanii* relative to *P. patula* inoculated samples from pathogen expression data.

## Supplementary Materials

The following are available online at https://www.mdpi.com/2076-2607/7/9/315/s1.
