# Supplementary material for: Dual RNA-Seq Analysis of the Pine-Fusarium circinatum Interaction in Resistant (Pinus tecunumanii) and Susceptible (Pinus patula) Hosts
Source: Microorganisms. 2019 Sep 4;7(9):315. doi: 10.3390/microorganisms7090315 (PMC6780516; doi:10.3390/microorganisms7090315)
Supplement: Supplementary file 1 [file microorganisms-07-00315-s001.zip › Supplementary_Figures.pdf]

Dual RNA-seq comparison of the pine-*Fusarium circinatum* interaction between resistant (*Pinus tecunumanii*) and susceptible (*Pinus patula*) hosts

Erik A. Visser[1], Jill L. Wegrzyn[2], Emma T. Steenkamp[1], Alexander A. Myburg[1], Sanushka Naidoo[1]*

[1]Department of Biochemistry, Genetics and Microbiology, Forestry and Agricultural Biotechnology Institute (FABI), Genomics Research Institute (GRI), University of Pretoria, Private bag X20, Pretoria 0028, South Africa

[2]Department of Ecology and Evolutionary Biology, University of Connecticut, Storrs, CT 06269, USA

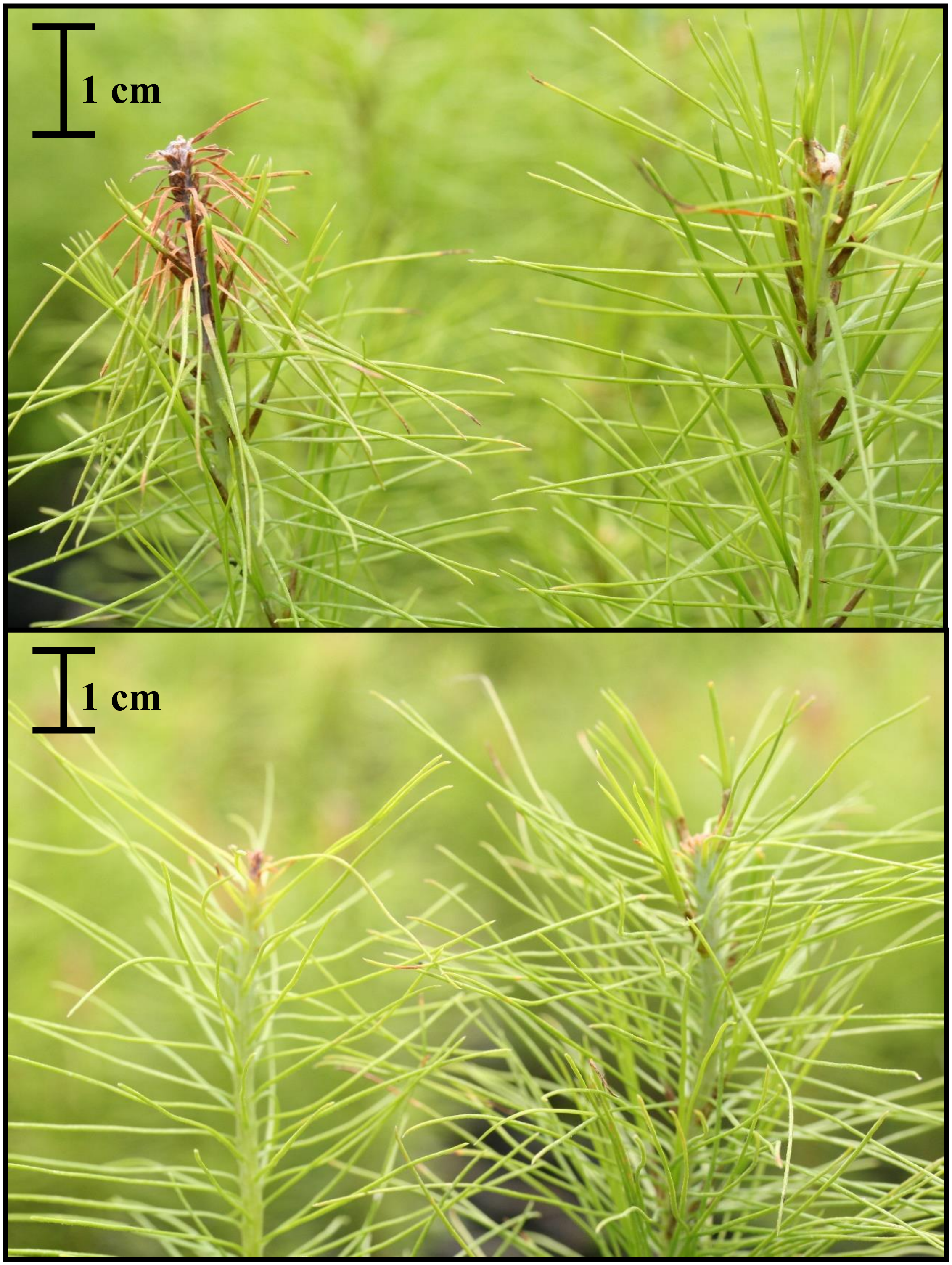

Figure S1 *Fusarium circinatum* disease symptoms on inoculated pine seedlings at 21 days post inoculation. Left – inoculated. Right – mock-inoculated. Top – *Pinus patula*. Bottom – *Pinus tecunumanii*.

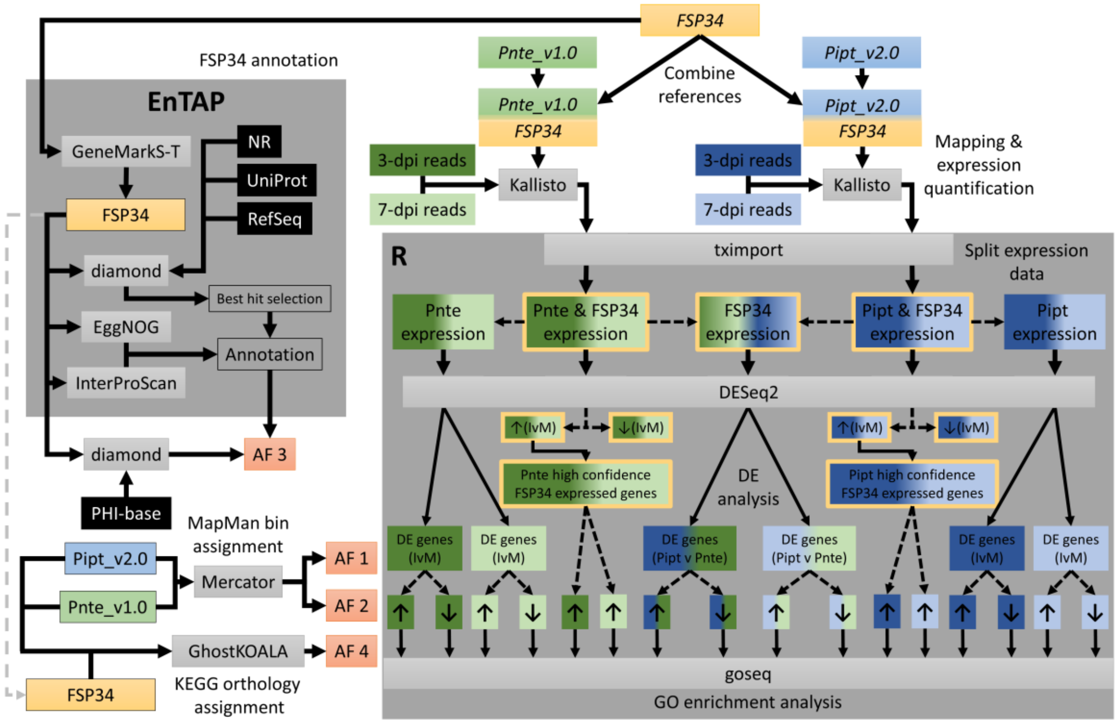

Figure S2 Bioinformatic methods flow diagram. FSP34 = *F. circinatum* proteome, Pipt_v2.0 = *P. patula* proteome, Pnte_v1.0 = *P. tecunumanii* proteome, in each case italics indicate transcriptomes. Grey boxes represent programs, orange boxes represent additional files (AF) and black boxes represent databases. Yellow represents *F. circinatum*, green represents *P. patula*, blue represent *P. tecunumanii*. In the R box dark colours represent 3-dpi and light colours represent 7-dpi. IvM = differentially expressed genes in inoculated relative to mock-inoculated samples, Pnte v Pipt = differentially expressed *F. circinatum* genes from *P. tecunumanii* data sets relative to *P. patula* data sets. Arrows refer to up- and down-regulated DE genes.

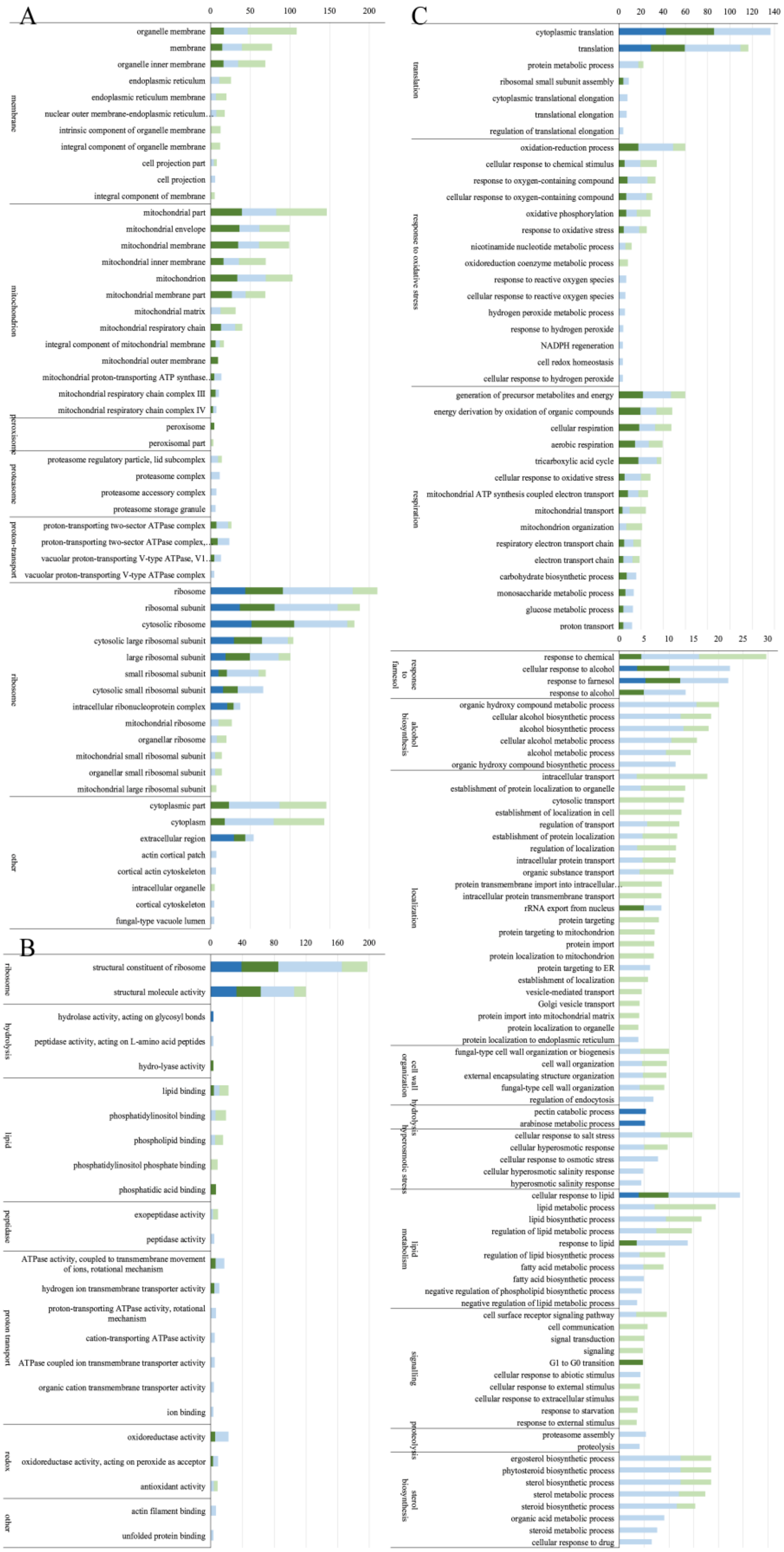
A
0 50 100 150 200
membrane
organelle membrane
membrane
organelle inner membrane
endoplasmic reticulum
endoplasmic reticulum membrane
nuclear outer membrane-endoplasmic reticulum...
intrinsic component of organelle membrane
integral component of organelle membrane
cell projection part
cell projection
integral component of membrane
mitochondrion
mitochondrial part
mitochondrial envelope
mitochondrial membrane
mitochondrial inner membrane
mitochondrion
mitochondrial membrane part
mitochondrial matrix
mitochondrial respiratory chain
integral component of mitochondrial membrane
mitochondrial outer membrane
mitochondrial proton-transporting ATP synthase...
mitochondrial respiratory chain complex III
mitochondrial respiratory chain complex IV
peroxisome
peroxisome
peroxisomal part
proteasome
proteasome regulatory particle, lid subcomplex
proteasome complex
proteasome accessory complex
proteasome storage granule
proton-transport
proton-transporting two-sector ATPase complex
proton-transporting two-sector ATPase complex,...
vacuolar proton-transporting V-type ATPase, V1...
vacuolar proton-transporting V-type ATPase complex
ribosome
ribosome
ribosomal subunit
cytosolic ribosome
cytosolic large ribosomal subunit
large ribosomal subunit
small ribosomal subunit
cytosolic small ribosomal subunit
intracellular ribonucleoprotein complex
mitochondrial ribosome
organellar ribosome
mitochondrial small ribosomal subunit
organellar small ribosomal subunit
mitochondrial large ribosomal subunit
other
cytoplasmic part
cytoplasm
extracellular region
actin cortical patch
cortical actin cytoskeleton
intracellular organelle
cortical cytoskeleton
fungal-type vacuole lumen
B
0 40 80 120 160 200
ribosome
structural constituent of ribosome
structural molecule activity
hydrolysis
hydrolase activity, acting on glycosyl bonds
peptidase activity, acting on L-amino acid peptides
hydro-lyase activity
lipid
lipid binding
phosphatidylinositol binding
phospholipid binding
phosphatidylinositol phosphate binding
phosphatidic acid binding
peptidase
exopeptidase activity
peptidase activity
proton transport
ATPase activity, coupled to transmembrane movement of ions, rotational mechanism
hydrogen ion transmembrane transporter activity
proton-transporting ATPase activity, rotational mechanism
cation-transporting ATPase activity
ATPase coupled ion transmembrane transporter activity
organic cation transmembrane transporter activity
ion binding
redox
oxidoreductase activity
oxidoreductase activity, acting on peroxide as acceptor
antioxidant activity
other
actin filament binding
unfolded protein binding
C
0 20 40 60 80 100 120 140
translation
cytoplasmic translation
translation
protein metabolic process
ribosomal small subunit assembly
cytoplasmic translational elongation
translational elongation
regulation of translational elongation
response to oxidative stress
oxidation-reduction process
cellular response to chemical stimulus
response to oxygen-containing compound
cellular response to oxygen-containing compound
oxidative phosphorylation
response to oxidative stress
nicotinamide nucleotide metabolic process
oxidoreduction coenzyme metabolic process
response to reactive oxygen species
cellular response to reactive oxygen species
hydrogen peroxide metabolic process
response to hydrogen peroxide
NADPH regeneration
cell redox homeostasis
cellular response to hydrogen peroxide
respiration
generation of precursor metabolites and energy
energy derivation by oxidation of organic compounds
cellular respiration
aerobic respiration
tricarboxylic acid cycle
cellular response to oxidative stress
mitochondrial ATP synthesis coupled electron transport
mitochondrial transport
mitochondrion organization
respiratory electron transport chain
electron transport chain
carbohydrate biosynthetic process
monosaccharide metabolic process
glucose metabolic process
proton transport
0 5 10 15 20 25 30
response to farnesol
response to chemical
cellular response to alcohol
response to farnesol
response to alcohol
alcohol biosynthesis
organic hydroxy compound metabolic process
cellular alcohol biosynthetic process
alcohol biosynthetic process
cellular alcohol metabolic process
alcohol metabolic process
organic hydroxy compound biosynthetic process
localization
intracellular transport
establishment of protein localization to organelle
cytosolic transport
establishment of localization in cell
regulation of transport
establishment of protein localization
regulation of localization
intracellular protein transport
organic substance transport
protein transmembrane import into intracellular...
intracellular protein transmembrane transport
rRNA export from nucleus
protein targeting
protein targeting to mitochondrion
protein import
protein localization to mitochondrion
protein targeting to ER
establishment of localization
vesicle-mediated transport
Golgi vesicle transport
protein import into mitochondrial matrix
protein localization to organelle
protein localization to endoplasmic reticulum
cell wall organization
fungal-type cell wall organization or biogenesis
cell wall organization
external encapsulating structure organization
fungal-type cell wall organization
regulation of endocytosis
hydrolysis
pectin catabolic process
arabinose metabolic process
hyperosmotic stress
cellular response to salt stress
cellular hyperosmotic response
cellular response to osmotic stress
cellular hyperosmotic salinity response
hyperosmotic salinity response
lipid metabolism
cellular response to lipid
lipid metabolic process
lipid biosynthetic process
regulation of lipid metabolic process
response to lipid
regulation of lipid biosynthetic process
fatty acid metabolic process
fatty acid biosynthetic process
negative regulation of phospholipid biosynthetic process
negative regulation of lipid metabolic process
signalling
cell surface receptor signaling pathway
cell communication
signal transduction
signaling
G1 to G0 transition
cellular response to abiotic stimulus
cellular response to external stimulus
cellular response to extracellular stimulus
response to starvation
response to external stimulus
proteolysis
proteasome assembly
proteolysis
sterol biosynthesis
ergosterol biosynthetic process
phytosteroid biosynthetic process
sterol biosynthetic process
sterol metabolic process
steroid biosynthetic process
organic acid metabolic process
steroid metabolic process
cellular response to drug

Figure S3 Gene ontology terms enriched among high confidence *in planta* expressed *F. circinatum* genes. (A) Cellular compartment, (B) Molecular Function and (C) Biological Process GO terms enriched within up-regulated data sets at 3- (dark bars) and 7- (light bars) dpi in *P. patula* (blue) and *P. tecunumanii* (green). The y-axes represent the absolute $\log_2$(q-value) following Benjamini & Hochberg False Discovery Rate (FDR) correction.
